# Predictors Associated with Health-Related Heat Risk Perception of Urban Citizens in Germany

**DOI:** 10.3390/ijerph17030874

**Published:** 2020-01-30

**Authors:** Sabrina K. Beckmann, Michael Hiete

**Affiliations:** Department of Business Chemistry, Ulm University, 89081 Ulm, Germany; sabrina.beckmann@uni-ulm.de

**Keywords:** heat risk perception, knowledge, heat wave, climate change, adaptation, health risks

## Abstract

The rising probability of extremely high temperatures and an increasing number of consecutive hot days caused by climate change—combined with the impact of these high temperatures on human health—is widely discussed in the literature. There are calls for the development of heatwave adaptation measures by governmental and scientific institutions. In this research, the predictors of health-related heat risk perception of urban citizens in Augsburg, Germany, were investigated. An online survey was conducted with 468 citizens, asking about their heat risk perception, knowledge about heat risks, and demographic data and health information. Statistical methods (Spearman correlation, unpaired *t*-test, ANOVA and multiple regression) were used to determine which factors were significant and relevant. The results show that the knowledge of heat risks, heat risk sensitivity and an external locus of control are the most important factors for heat risk perception. The health implication score and chronic disease show significant effects in descriptive statistics. Furthermore, younger people showed the highest heat risk perception of all age groups. Surprisingly, income, education, living alone and gender did not play a role in heat risk perception. The findings imply a need for better and intensified heat risk communication in urban areas—especially among elderly people—and thus are important for creating acceptance towards heat wave risks, which is a prerequisite of willingness to adapt.

## 1. Introduction

The rising probability of extremely high outdoor temperatures, more consecutive hot days and of droughts in the coming years has already been demonstrated by international research in the last decade [1]. The extreme heatwaves in Europe during summer 2003, which caused 70,000 people to die prematurely [2], as well as the hot summers in 2018 and 2019, which caused record temperatures all over Europe [3] fostered interdisciplinary research worldwide about the impact of heat on human health and possible adaptation measures. Studies have shown that heat is directly linked to higher mortality and infectious diseases, especially in cities with a high population density; this then triggers the existence of urban heat islands (UHIs) [4,5,6,7,8]. UHIs are caused by increased heat absorption and the capture of heat by paved environments and concrete buildings during daytime and a slow heat release in the night [9]. This slow temperature decline is a problem for the inhabitants of a city, as shown in a study from eastern India, where heatwaves are causing immense health problems. There, the mortality risks rose by 2% for every degree above 36.2 °C [10]. Heatwaves have been found to not only induce physical but also psychological health impacts, such as anxiety, depression and aggression, having diverse effects on different community groups [11,12].

In Europe, the most dramatic *rise* of temperature is projected to occur in Central and South-east Europe, with Mediterranean heatwaves expected to occur in these regions [13,14]. However, the people living in these regions do not have experience dealing with heatwaves as the people in more affected areas do; this causes a higher vulnerability and a need for adaptation measures and risk awareness. While between 2008 and 2017 the number of days of illness among employees in Germany because of heat doubled [15], by 2050, the temperatures in Berlin are expected to be equal to the current temperatures of some Australian cities [16], which most likely will cause a rising number of sick days. Reid et al. [17] criticised the fact that most of the studies use data that are applicable to a whole nation rather than just a small region because of the varied premises given for vulnerability factors, resulting in different possible applicable adaptation measures.

Hence, the current paper focuses on a single town, Augsburg, a German city located 70 km northwest of Munich. Augsburg is usually only moderately exposed to extreme temperatures. However, because the frequency of hot days during summer months is expected to increase, citizens there are more and more affected by its consequences, and the awareness of health risks caused by heat waves needs to be elevated to establish resilience against these consequences.

In the literature, there is a general call for the development of urban resilience against heat waves [18]. It is known that a system’s resilience increases with its adaptive capacity [19]. Therefore, to become resilient against the impacts of heat waves, urban citizens must develop heat adaptation measures [19]. Liu et al. [20] found that the higher the perception of a potential risk in general, the higher an individual’s willingness to adapt will be. In contradiction to the already investigated factors influencing heat stress perception (e.g., the belief of being helplessly subjective to heat, working outside, being a migrant worker or cognitive performance pressure at work) [21,22,23], the research on heat risk perception in Germany is scarce. In the literature, there are some factors associated with heat risk perception for other countries and regions. For example, in Phoenix, Arizona, the perception of heat risk is higher among Hispanics; among women and young people [24]; among vulnerable groups, people with one or more chronic diseases or of low income [25]; among those married [26]; and among those suffering from more health impacts during heat [27]. Furthermore, it is known that the perception of heat risks can also depend on political orientation and climate change beliefs [28]; being part of an ethnic minority; the amount of time working outside [29]; and people’s subjective health status [30]. The results show that the factors associated with heat risk perception are divergent and depend on the region, its climate conditions and the demographic factors of the people participating.

Therefore, in the current study, factors from previous studies were selected (Table 1) for reanalysis to answer the following research question: which predictors statistically determine Augsburg’s citizens’ health-related heat risk perception? By answering this question, the present paper contributes not only to the growing literature on the factors driving heat risk perception, but also to the development of resilience against heat in German cities and their heat-related inexperienced inhabitants by examining the following:

1. Identifying the factors that statistically determine heat risk perception among urban citizens in Germany, specifically Augsburg, which influences their willingness to adapt to heat;
2. Highlighting the gap between scientific heat risk perception and citizens’ heat risk perception, suggesting potential ways to begin adaptation measures.

## 2. Materials and Methods

A questionnaire survey was conducted in July 2019 in Augsburg, Germany. Augsburg has nearly 300,000 inhabitants and covers an area of 147 km^2^. The annual mean temperature was 8.5 °C for the period 1981–2010, with July being the hottest month, with 18.1 °C, and August, with 17.5 °C, the second hottest; in addition, there are six hot days per year, that is, days with maximum temperatures above 30 °C [31]. However, the number of hot days in recent years has risen. In 2018, as well as 2019, there were 12 hot days (2017: 11 hot days, 2016: eight hot days). The hottest day in 2019 was on June 26: 34.6 °C. During the survey period from July 1, 2019 to September 1, 2019, the highest temperature was 34.4 °C, measured on July 25 [32]. The survey was conducted within the frame of the project “Augsburg stays cool” [33]. The project is funded by Bundesministerium für Umwelt, Naturschutz und nukleare Sicherheit (Federal Ministry for the Environment, Nature Conservation and Nuclear Safety) and was conducted over a period of two years. Besides Ulm University being the coordinator of the project and the city of Augsburg the practice partner, the bifa GmbH (environmental institute), Augsburg University, the consulting office SLU, specialising in analysing remote sensing data, and the German Aerospace Centre DLR were also involved. The general aim of the research project was to identify heat hotspots in the city, raise awareness of heat risks among citizens and develop heat adaptation measures for the urban area. In the survey, questions were raised regarding heat risk perception and knowledge of heat risks, but also regarding sociodemographic details such as age, education, gender and personal information, such as chronic diseases or health-related problems during heatwaves. Data collection took place in July 2019 among participants who agreed to place a temperature data logger in their bedrooms to measure indoor temperatures during summer 2019. The research area was defined by dividing the city into local climate zones (LCZs). A cross-section was drawn over the city to ensure the inclusion of as many LCZs as possible. People living in the chosen area received an invitation to participate in the project and were able to sign up to receive the temperature data logger and access to the online survey; the participants could alternatively have chosen a telephone interview. In this manner, 468 questionnaires were completed. Note that the measured temperature data are not part of the current paper.

The constructs used in the survey were based on previous questionnaires, e.g., [34,35]. To find out about heat risk perception and its relation to the perception of other natural hazards, the items from Martens et al. [36] were used, who constructed the instrument based on the research by Becker [37] and Rosenstock [38]. The items can be found in the GESIS database of the GESIS—Leibniz Institute for the Social Science [39]—and have already been tested and validated. Two of the eight statements were modified to ask about a) heat risk perception for the individual and b) for the environment. Knowledge about heat risks was operationalised in 10 statements about heatwave risks; these statements were partly adopted from [34] and partly completed by statements taken from the information in Bunz and Mücke [40]. Furthermore, the locus of control of reinforcement of the individuals was investigated, which here is defined as a generalised expectation of internal or external reinforcement. The internal locus of control describes the extent to which participants can control events happening in their lives and experience them as a consequence of their own behaviour. The external locus of control is defined as the extent to which an individual thinks everything happening in his or her life depends on destiny or fate, or is under the control of others [41,42,43]. The construct plays an essential role in predicting and explaining a person’s behaviour and—in this case—the conviction to act against heatwaves and, therefore, the willingness to take adaptation measures. For reproduction purposes—and for a better relation to the survey elements—the statements used in the questionnaires have been translated into English and can be found in Supplementary Material S2.

For data analysis, IBM SPSS^®^ [44] was used. To get an overview of the participants’ knowledge and health-related heat risk perceptions, a descriptive analysis was conducted, followed by bivariate correlations, one-way ANOVAs, unpaired *t*-tests and, finally, a regression model, to identify the predictors of health-related heat risk perception [45]. All normally distributed groups were first tested for the absence of data homogeneity. Sociodemographic variables such as gender, age and income—but also personal health information such as chronic diseases or health problems during heatwaves—were tested. The individual’s locus of control was also taken into account as a predictor variable. Table 2 summarises the relevant variables with their categories and the scales used for statistical analysis.

Some of the variables have been transformed into scores to make them usable for statistical testing (see Table 2). To ask about risk perception, we used eight statements about four different natural hazards. For each statement, we asked about 1) the risks for the individuals and 2) the risks for their environment (e.g., ‘I think that heat waves put my health in danger’; ‘Heat waves are dangerous for plants and animals’). The statements were measured on a 4-point Likert scale ranging from 1 (I don’t agree) to 4 (I fully agree). To determine the heat risk perception, the mean of both statements for the individual and the environment was taken. The final scale for heat risk perception ranged from 1 (very weak heat risk perception) to 4 (very strong heat risk perception). The knowledge score was determined by following [34]. The element was operationalised in 10 statements about heat wave risks, which the participants were able to answer with ‘correct’, ‘incorrect’ or ‘I don’t know’. Each correct answer to a statement was coded with 1 and wrong answer or statement ‘I don’t know’ with 0. The answers were then dichotomised to a knowledge score that ranged from 0 (no knowledge at all) to 10 (very high knowledge). The internal and external locus of control was tested using the items in [41]. The participants were asked to answer the four statements on a 5-point Likert scale, which were then added and classified into low, middle and high. The external locus of control was classified as low (2–4), middle (5–7) and high (8–10), determining the degree of the participant’s conviction that their lives depend on other people, fate or accidents. The internal locus of control was divided into scores of low (4–6), middle (7–8) and high (8–10), depicting the degree of the participant’s conviction that their lives are in their own hands and that they can control everything happening. The difference between the classification of scores can be explained by the size of each group, hence ensuring a standard distribution of data. For health implications, seven possible health problems during heat found in the literature [40,46] (drowsiness, sleeping problems, headache, concentration problems, vertigo, nausea and cardiovascular problems) were proposed. The score was developed using the mean of the seven possible health implications (0 = never, 1 = sometimes, 2 = often). This resulted in a score ranging from 0 to 2. The scores were then categorised into 0 (no health implication during heat), 0.1–1.0 (moderate health implication during heat) or 1.1–2.0 (high health implication during heat).

## 3. Results

This section first introduces the descriptive statistics of the data, showing the distribution of heat risk perception, the risk perception of alternative natural hazards, knowledge about heat risks and participant subjective sensitivity. Then, the findings of the statistical analysis regarding the factors associated with heat risk perception, followed by the results of the factors associated with knowledge about heat risks, are shown.

### 3.1. Descriptive Statistics

The distribution of education among the participants is shown in Figure S1 (all figures are part of Supplementary Material S1). For the current study, 59.4% of the 468 participants were female, and 37.2% were living alone. At 59.2%, most held a university degree, and 61.1% were between 30 and 64 years old, 23.5% between 18 and 29 years and 15.4% older than 65. Of all the participants, 28.2% answered that they were currently not employed, including retired people. In addition, 19.7% of the participants were suffering from a chronic disease, with 2.8% preferring not to give an answer to this question. Figure S2 shows that most (48.7%) already considered heat as a problem in their city and in their home. Heat in the neighbourhood was considered a problem by 38.3% of the participants. Ninety-one participants (19.4%) considered heat is neither now, nor will be in the future (by 2030), a problem in the neighbourhood, whereas for the city, there were only 33 people (7.1%) answering that heat is neither a problem now nor will be in the future. Figures S3 and S4 show the distribution of the perception regarding heat risks and risks of alternative hazards such as water and air pollution and the greenhouse effect. Figure S4 shows that more participants fully agreed that heat waves pose more of a risk than other natural hazards; the subjective heat sensitivity is shown in Figure S5. The participants were able to answer the question ‘How heat sensitive would you rate yourself?’ with ‘not at all’, ‘rather not’, ‘neutral’, ‘rather’ or ‘very much’. Most participants (72%) answered that they consider themselves to be rather or very strongly heat sensitive. Only 18.8% would rate themselves as rather not or not heat sensitive at all. Regarding the knowledge about heat risks, the participants showed rather high knowledge, as shown in Figure S6. Here, 422 (90.2%) of the participants gave the correct answer to five or more statements about heat risks, and only nine people answered nine or more statements incorrectly. Finally, Figure S7 shows the amount of health implications during heat. The most common health problems occurring during heat are shown to be drowsiness (84% of the participants claiming to experience drowsiness sometimes or often during heat), sleeping problems (80% of the participants suffering sometimes or often from sleeping problems during heat) and concentration problems (77% of the participants sometimes or often having problems with their concentration during heat). Furthermore, over half of them (58%) reported sometimes or often having headaches during heat, and 48% sometimes or often experiencing vertigo. In addition, 39% reported having cardiovascular problems and 18% trouble with nausea.

### 3.2. Factors Associated with Heat Risk Perception

The Spearman’s correlation coefficients show a moderate correlation between heat risk perception and an individual’s subjective heat sensitivity, which is followed by a weak correlation with the health implication score. There is a weak correlation between the knowledge about heat risks and heat risk perception, as well as a weak correlation between the external locus of control and heat risk perception. These results are presented in Table 3. Correspondingly, Table 3 shows a very weak negative correlation between the internal locus of control with heat risk perception, meaning that the more participants think their lives are controlled by them, the lower their heat risk perception is.

The significant test statistics regarding the relevant variables are listed in Table 4, and an applicable regression model is presented in Table 5. It is important to note that people with higher knowledge were more likely to perceive heat as a risk, shown in Table 4 and Table 5; this result aligns with O’Riordan, who assessed this effect in 1986 in general with risk perception and education about risks [47]. The ANOVA shows a significant difference among the age groups regarding heat risk perception. Although the group of 65–74 years of age had the lowest heat risk perception, followed by participants older than 74 years, heat risk perception was the highest among people from 18–29 years of age. This effect cannot be found in the regression model in Table 5 but is still worth keeping in mind for future adaptation measures. Furthermore, the descriptive statistics show that people suffering from chronic diseases, people with more health problems during heat and people calling themselves subjectively heat sensitive were more likely to perceive heat as a risk. However, the regression model in Table 5 only shows significance for subjective heat sensitivity. The third predictor of heat risk perception, which has a significant effect in the regression model, is the external locus of control (people who tend to think their life is controlled by others or depends on fate) and shows a higher risk perception. Here, there were some predictor variables tested in the descriptive statistics that did not show any significant effects. These variables are gender, employment status and education. Furthermore, there was no statistically significant effect among people living alone and people not living alone. Income and bedroom orientation did not give significant results.

## 4. Discussion

The survey conducted in Augsburg shows that almost half (48.7%) of the 468 participants already saw heat as a problem in their city and homes. More participants fully agreed with the risks caused by heat waves compared with the risks caused by other natural hazards. Most of the participants showed rather high knowledge, with 90.2% of them giving the correct answer to five or more of the statements. Nearly three quarters of the participants (72%) considered themselves to be rather or very strongly heat sensitive.

The Spearman’s correlation coefficients, the ANOVA and the regression model show significant results for heat risk perception associated with the knowledge score. This is a very important result and is helpful in the development of adaptation measures. In the descriptive statistics, there is a significant effect of the health implication score on heat risk perception. This result aligns with [25], [29], [27] and [30], who found that sick people or people with a poor subjective health status show a higher risk perception towards heat or towards climate change. However, it is interesting to see that the linear regression model in the current study did not show a significant effect of the health implications. The same applies for chronic diseases, for which the *t*-test showed an effect on perceived heat risks, supporting the findings of [25] and [29]; however, chronic disease is not a significant factor in the regression model. Furthermore, it was not possible to verify the findings of [25] that show that older people have a higher risk perception. The descriptive analysis in the current study shows the opposite: younger people (18–24 years of age) showed the highest heat risk perception, which aligns with the findings of [24]. Even though the regression model did not show a significant effect for the different age groups, it is important to keep in mind that the youngest age group showed the highest risk perception, while the group of 30–64 years of age showed the lowest risk perception.

Another significant factor in relation to heat risk perception is subjective heat sensitivity. Here, a moderate correlation was found, showing that the more participants considered themselves to be heat sensitive, the more they perceived heat as being a risk. The effect of subjective heat sensitivity on heat risk perception can be found in the regression model in Table 5. One factor that has not been researched in the literature thus far is the internal and external locus of control. The findings of both the ANOVA and regression model show that the higher an individual’s external locus of control is, the higher his or her risk perception towards heat will be. Hence, the more a person is convinced that the incidents happening in their life are based on fate or accident, the more they perceive heat as being a risk. In the current study, income did not significantly correlate with heat risk perception, whereas the authors of [26] and [25] found income to be a significant factor for risk perception while also finding being married, and therefore not living alone, as significant. In our study, neither living alone nor income showed significant results. Even when the significance of the income level excluding the youngest group of participants (18–24 years of age) was tested—here because this age group mainly contains students with low income—it did not show significance. Neither did gender, employment status, education nor bedroom orientation show any significant association with knowledge or heat risk perception.

The rising probability of more heat waves in the future and the lack of experience of urban citizens in Germany in dealing with these heat waves causes a higher vulnerability and, therefore, a need for adaptation measures and more awareness. The focus on Augsburg, as an urban area in southern Germany, is important for taking into consideration regional premises and differences compared with other countries or climate regions. By identifying the predictors associated with heat risk perception, the current paper contributes to the development of heat resilience in German cities. It is important for future adaptation measures to better understand heat risk perception and the factors associated with it.

The main limitation of the current research is that it was only conducted in one German city. The sample size of 468 participants cannot be seen as representative of the whole population of the city. In addition, the survey took place in one summer over a period of one month of heat but not during steadily high temperatures. Therefore, the judgement of the participants could have been influenced by extreme heat at the moment of participation. Furthermore, the survey was conducted in German, and the results were translated into English for this publication. Finally, not all the participants took part in the survey online; some of them were interviewed over the phone because they did not have access to the Internet. The items used in this survey were not completely taken from former scientific publications, because some of them were modified to fit the research design in a German city. As a result, not all of the results can be directly compared with the results of other studies.

## 5. Conclusions

In the future, the increasing number of consecutive hot days in Central Europe will lead to a greater necessity for heat adaptation measures, especially among urban citizens, who belong to the most vulnerable groups. The aim of the current paper was to determine which factors are statistically associated with heat risk perception among urban citizens in Augsburg, Germany. One of the key strengths of the current study is that it is the first to investigate the factors associated with heat risk perception among urban citizens in a city in southern Germany. The findings are the first step for the further comparison of similar cities and the development of heat risk perception over time. This is crucial because high temperatures are directly linked to higher mortality and physical and psychological diseases, and people must take adaptation measures to prevent their health being harmed.

The most important finding is that knowledge and subjective heat sensitivity are directly correlated with heat risk perception, meaning people who know more about heat waves are more likely to perceive heat as a risk and take adequate adaptation measures. Without knowledge about the possible risks and awareness among people about their health being threatened by heat, they might not be willing to accept heat as a risk and take sufficient actions to adapt to extreme heat. To adequately address these factors when implementing adaptation measures, future research should analyse which factors influence knowledge of heat waves and subjective heat sensitivity, even though part of the heat risk perception cannot be influenced, as an individual’s external locus of control was identified as a significant factor.

The findings imply a need for intensified communication of heat risks in urban areas and are important for creating acceptance of heatwave risks, which can lead to willingness to adapt. The current study also recommends communication, especially among elderly citizens between the ages of 64 and 74, because they are the most vulnerable group and the group with the least risk perception. It is important to know that people with chronic diseases and a high health implication score are already sensing the risks of heat. However, it should not be taken for granted that those groups always seek information themselves. Communicating information to them as well should also be considered. The study also shows that gender, high education level and income level do not play a role when it comes to heat risk perception in German cities. Finally, although the findings are statistically correct, there are also young people with a low risk perception who would benefit from better communication.

## Figures and Tables

**Table 1:** Selection of variables to be included.

Factors Identified	Ref.	Statement
Hispanics	[24]	Not applicable in sample
Women	[24]	Included
Young people	[24]	Included
Vulnerable groups	[25]	Has been included and itemised in various sub factors (age, living situation, income, etc.)
Chronic diseases	[25]	Included
Low income	[25]	Included
Being married	[26]	Has been substituted in the study by ‘not living alone’
Suffering health impacts during heat	[27]	Included
Political orientation	[28]	Political orientation in the USA not applicable for Germany
Climate change beliefs	[28]	Substituted by item ‘heat seen as a problem’
Ethnic minority	[29]	Not applicable in sample
Time working outside	[29]	Excluded, study focusses on temperatures at home
Subjective health status	[30]	Not necessary because health implications during heat are asked; substituted by subjective heat sensitivity

**Table 2:** Variables and scores included in the data analysis.

Variables	Category	Scale
Heat risk perception		1 (very weak)—4 (very strong)
Knowledge of heat risks		0 (none at all)—10 (very high)
Health implications score	Drowsiness Sleeping problems Concentration problems Vertigo Headache Nausea Cardiovascular problems (Summarised to one score)	0 (none) 0.1—1 (moderate) 1.1—2 (high)
Locus of control	Internal External	1 (low) 2 (middle) 3 (high)
Subjective Heat Sensitivity		1 Not at all (sensitive) 2 Rather not 3 Neutral 4 Rather 5 Very much
Heat as a problem	In my city In my neighbourhood In my house	1 (already today) 2 (maybe in future) 3 (neither today, nor in future)

**Table 3:** Spearman’s correlation for heat risk perception.

Spearman’s rho	Heat Risk Perception
Heat risk perception	.
Knowledge about heat risks	0.273 ***
Health implication score	0.351 ***
Subjective heat sensitivity	0.423 ***
Internal locus of control	−0.136 ***
External locus of control	0.169 ***

***. Correlation is significant at the 0.05 level (two-tailed).

**Table 4:** Differences in heat risk perception.

Variable	Category	*N*	Heat Risk Perception	
			Mean	Test Statistics
			Mean Rank ^a^	
Knowledge about heat risks	0—3 (low)	21	156.64	F^a^ = 24.819 ***
	4—7 (moderate)	314	221.15	
	8—10 (high)	133	278.31	
Age group	18—29	110	2.94	F = 3.252 **
	30—64	286	2.89	
	65—74	42	2.54	
	Older than 74	30	2.85	
Chronic disease	Yes	92	3.05	*t* = 2.5 **
	No	363	2.81	
Health implications score	None	29	2.241	F = 33.531 ***
	Moderate	332	2.247	
	High	107	2.852	
Subjective heat sensitivity	Not at all	24	2.23	F = 25.456 ***
	Rather not	64	2.34	
	Neutral	43	2.55	
	Rather	218	2.87	
	Very strong	119	2.85	
Internal locus of control score	Low	27	2.91	F = 2.432 *
	Middle	146	2.97	
	High	295	2.79	
External locus of control score	Low	296	2.77	F = 6.745 **
	Middle	153	2.94	
	High	19	3.39	

^a^ = Kruskal Wallis based on mean rank was used because the standard distribution for group 0—3 (low) is not given; F^a^ = Kruskal Wallis Test; F= ANOVA; *t* = unpaired *t*-test. * Significant at the 0.1 level. ** Significant at the 0.05 level. *** Significant at the 0.001 level.

**Table 5:** Factors of heat risk perception (multiple regression).

Model Summary	R^2^ = 0.264, R^2^_adj_ = 0.253, F = 22.933, *p* < 0.001			
Dependent Variable ^a^	Unst. β	Std. Error	Std. Coefficient β	*t*
Knowledge about heat waves	0.129	0.021	0.257	6.256 ***
Age group				ns
Chronic disease				ns
Subjective sensitivity	0.256	0.032	0.354	7.881 ***
Health implication score				ns
Internal locus of control				ns
External locus of control	0.066	0.023	0.123	2.828 **

^a^ = Dependent variable: Heat risk perception; method: Enter; ns = not significant; Unst. β = Unstandardised Beta; Std. Error = Standard Error; Std. Coefficient β = Standardised Coefficient Beta. ** Significant at the 0.05 level; *** Significant at the 0.000 level.

## Supplementary Materials

The following are available online at https://www.mdpi.com/1660-4601/17/3/874/s1, Supplementary Material S1: Figures of descriptive analysis of data; Supplementary Material S2: English versions of reproduction purposes, a better relation to the survey elements, and the statements used in the questionnaires; Supplementary Material S3: Detailed survey data in Excel.
