# Supplementary material for: Predictors Associated with Health-Related Heat Risk Perception of Urban Citizens in Germany
Source: Int J Environ Res Public Health. 2020 Jan 30;17(3):874. doi: 10.3390/ijerph17030874 (PMC7038119; doi:10.3390/ijerph17030874)
Supplement: Supplementary file 1 [file ijerph-17-00874-s001.zip › BeckmannHiete_S1.pdf]

Supplementary Material S1: Figures

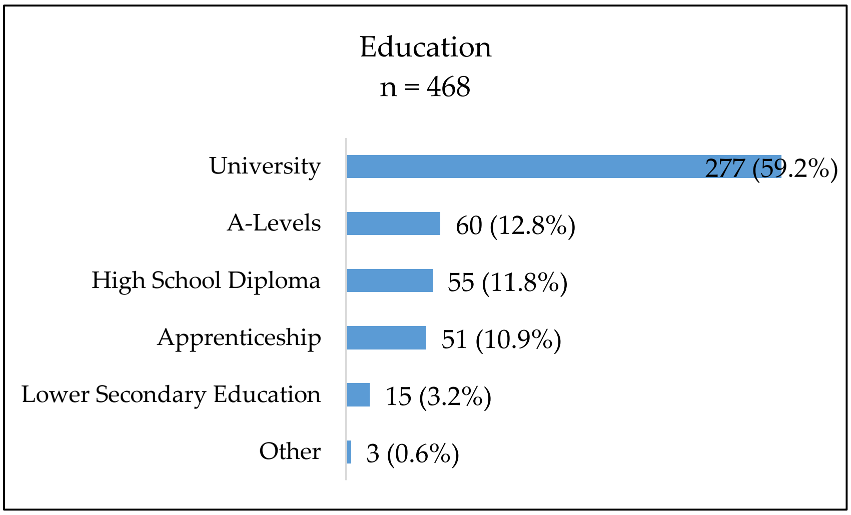

Figure S1 Education of participants

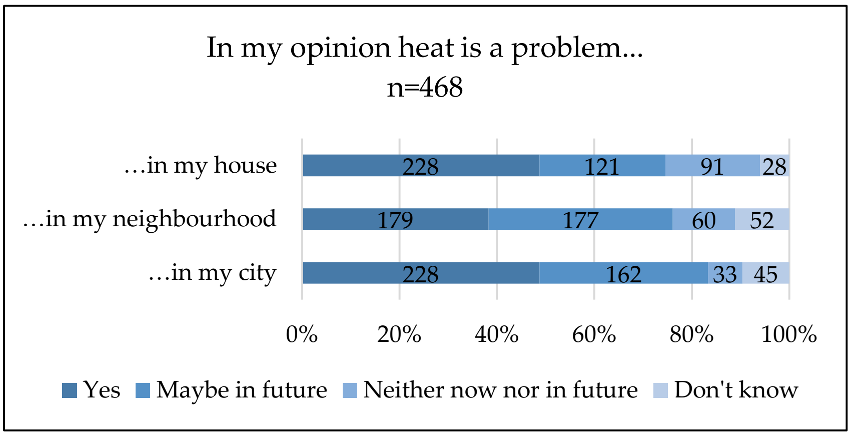

Figure S2 Heat as a problem in house, neighbourhood and city

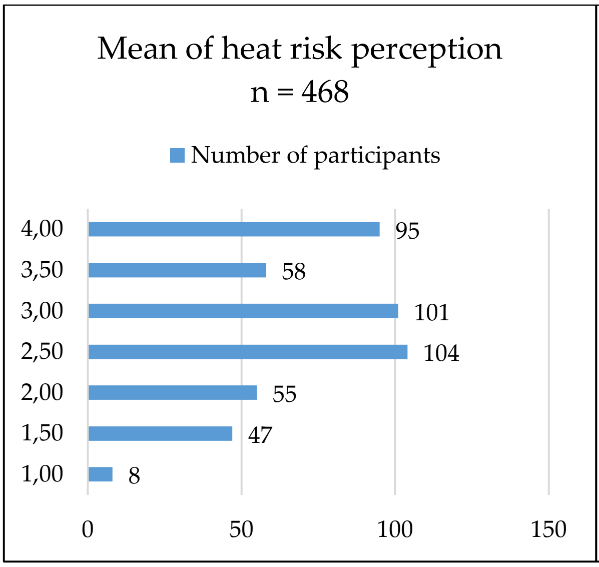

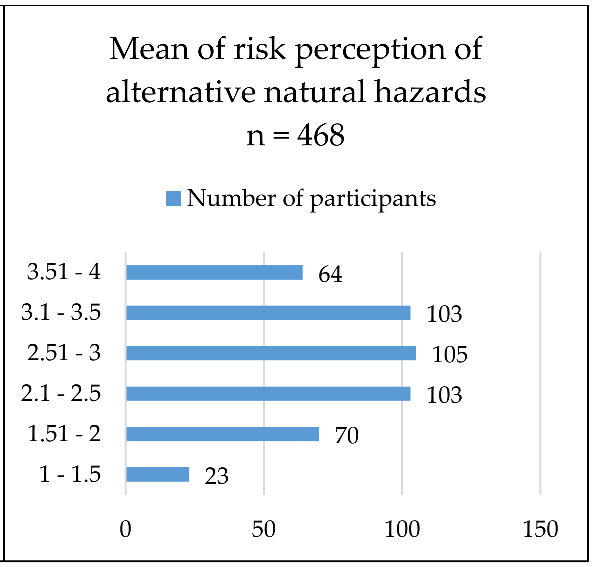

Figure S3 Mean of risk perception of alternative natural hazards

Figure S4 Mean of heat risk perception

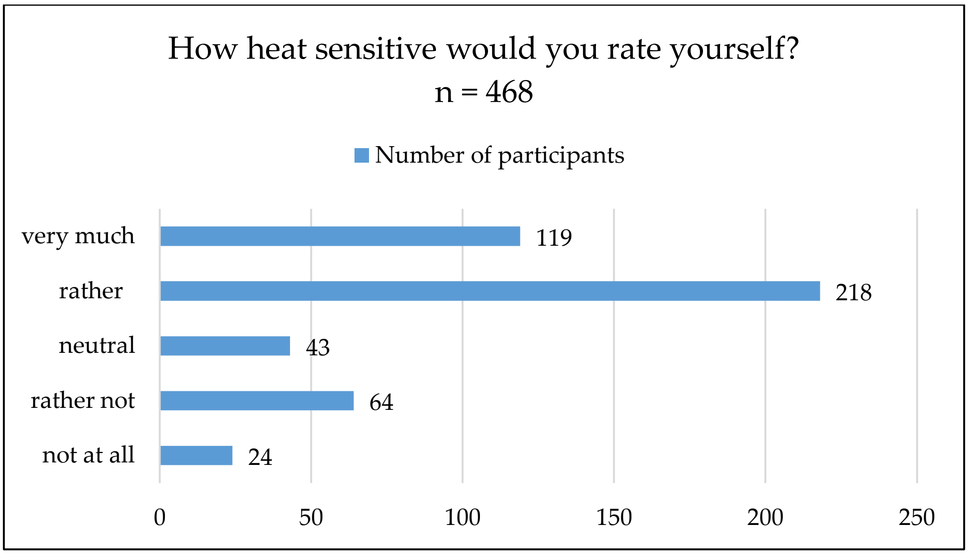

Figure S5 Subjective heat sensitivity

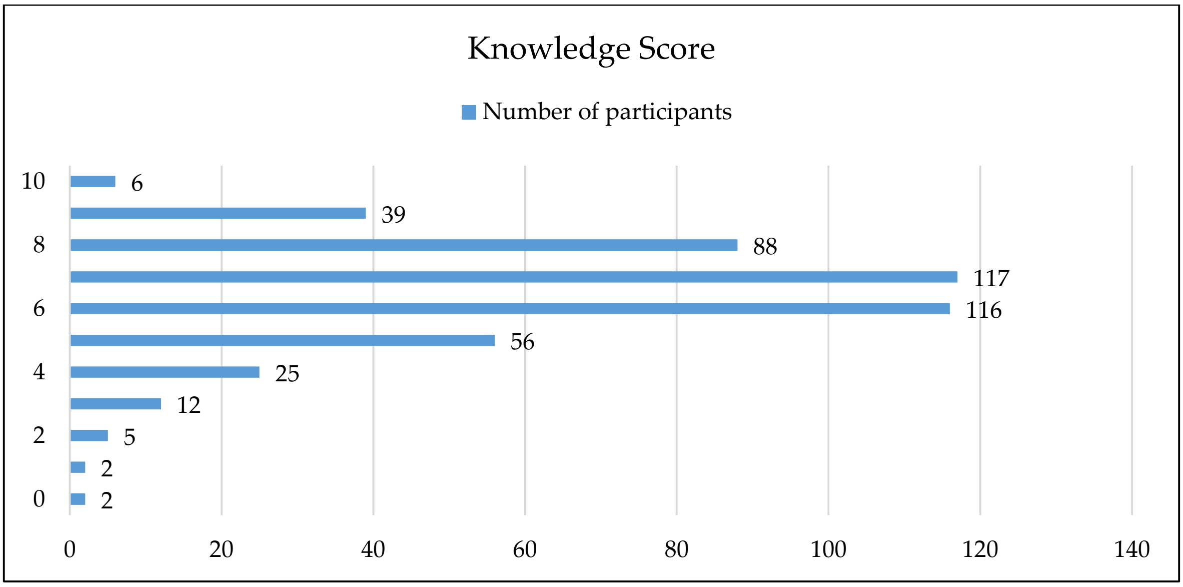

Figure S6 Knowledge Score

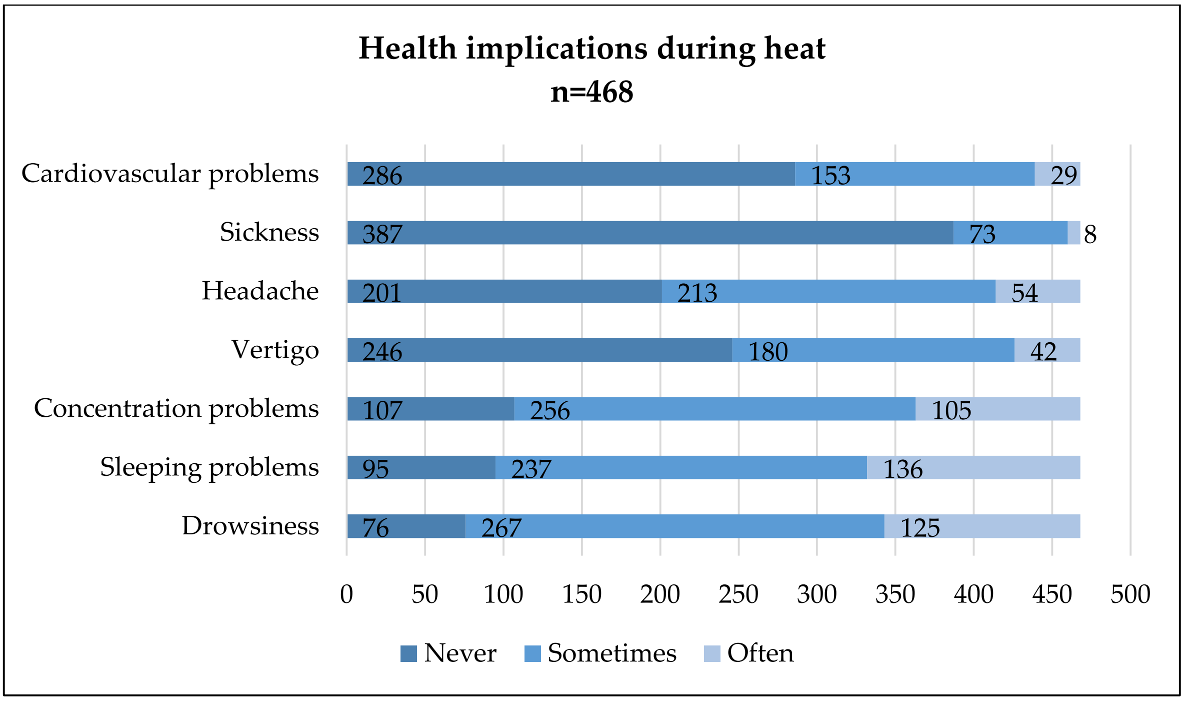

Figure A7 Reported health implications during heat
