# Supplementary material for: Predictors Associated with Health-Related Heat Risk Perception of Urban Citizens in Germany
Source: Int J Environ Res Public Health. 2020 Jan 30;17(3):874. doi: 10.3390/ijerph17030874 (PMC7038119; doi:10.3390/ijerph17030874)
Supplement: Supplementary file 1 [file ijerph-17-00874-s001.zip › BeckmannHiete_S2.pdf]

Supplementary Material S2

For reproduction purposes, and for a better relation to the survey elements, the statements used in the questionnaires have been translated into English and can be found in this document.

Table S1 Statements about risk perception

Item No.	Statement[a]
1	I feel my health is threatened by the increasing pollution of drinking water
2	Animals and plants are endangered due to water pollution
3	I think that heat waves endanger my health
4	Heat waves threaten plants and animals
5	I am afraid that I become sick by air pollution
6	Air pollution is a serious threat to plants and animals
7	The greenhouse effect probably has bad consequences for myself.
8	The greenhouse effect puts plants and animals to danger.

[a] = Four-level response scale: I agree; I agree to a large extent; I partly agree; I do not agree.

Table S2 Knowledge about heat waves

Item No.	Statement[a]
1	Older and very young people are particularly vulnerable during a heat wave.
2	Excessive sweating during a heat wave may be a sign of heat stress.
3	People with a cardiac disease are in danger of becoming sick during a heat wave.
4	Heat-related diseases can lead to death.
5	There is no such evidence for heat waves to cause respiratory diseases.
6	Diabetes is an example for a heat-related disease.
7	Heat waves foster the development of harmful bacteria in water and food.
8	Heat waves can be a factor for depression and anxiety.
9	Due to the building's shade, heat waves are less common in cities than in rural areas.
10	Heat stress during nighttime is worse than heat stress during daytime.

[a] = Response selection: true; false; I don't know.

Table S3 Internal and external locus of control

Item No.	Statement[a]
1	I am my own boss.
2	If I work hard, I will succeed.
3	Whether at work or in my private life: What I do is mainly determined by others.
4	Fate often gets in the way of my plans.

[a] = Five-level response scale: I totally agree (1) to I do not agree at all (5)
